# The Effect of Mindfulness-Based Stress Reduction Counseling on Blood Glucose and Perceived Stress in Women with Gestational Diabetes

**DOI:** 10.1055/s-0043-1775810

**Published:** 2023-10-16

**Authors:** Moslemi Zadeh Zeinabeh, Ahmadi Atefeh, G. Hazanfar Pour Masumeh, Dehesh Tania, Sanjari Mojgan, Alidousti Katayoun

**Affiliations:** 1 Kerman University of Medical Sciences, Kerman, Iran

**Keywords:** perceived stress, mindfulness-based stress reduction, gestational diabetes, blood sugar

## Abstract

**Objective** Gestational diabetes can cause maternal and neonatal morbidity. Psychological factors, especially stress, play a meaningful role in diabetes management. Therefore, the present study aimed to investigate the effect of Mindfulness-Based Stress Reduction counseling on blood sugar and perceived stress in women with gestational diabetes.

**Methods** The present quasi-experimental interventional study was performed on 78 women with gestational diabetes. In the intervention group, a Mindfulness-Based Stress Reduction counseling program was conducted by the researcher in 8 sessions of 90 minutes twice a week. The Cohen stress questionnaire was filled in both groups. Also, fasting blood sugar and 2-hour blood sugar levels were measured in both groups. Statistical analysis was performed using the independent T-Test, the paired T-Test, the Mann-Whitney and Wilcoxon Tests using IBM SPSS Statistics for Windows version 20 version (IBM Corp., Armonk, NY, USA).

**Results** The mean age of pregnant women in the intervention group was 28.84 ± 6.20 years old and 29.03 ± 5.42 years old in the control group. There was a significant mean difference between the fasting blood sugar score ( *p* = 0.02; - 6.01; and - 11.46) and the 2-hour fasting blood sugar score ( *p* < 0.001;12.35; and - 5.3) and the perceived stress score ( *p* < 0.001; 35.57; and - 49.19) existed between the intervention and control groups after the intervention.

**Conclusion** The results of the present study showed that mindfulness-based stress reduction counseling is effective in reducing blood sugar levels and reducing perceived stress in women with gestational diabetes treated with diet.

## Introduction

Diabetes is defined as a chronic metabolic disorder affecting carbohydrates, proteins, and fats, which is caused by a deficiency or resistance to insulin and hyperglycemia. Gestational diabetes is a disorder of glucose tolerance which is first started or diagnosed during pregnancy and is the most common metabolic disorder during pregnancy that can predispose the mother and fetus to serious and fatal complications. Children of women with gestational diabetes appear to be at increased risk for obesity and glucose intolerance and have a greater risk of developing diabetes in late adolescence. Moreover, the risk of fetal malformations is 4 to 10 times more common in mothers with uncontrolled gestational diabetes. 1 The prevalence of gestational diabetes in the world is ∼ 1 to 14% of all pregnancies. 2 Among many studies that have been done in recent years on the etiology, course, prognosis, and treatment of diabetes, psychological factors have received special attention. One of the most important psychological factors affecting the incidence of physical health disorders, such as diabetes, is stress. 3 Stress is defined as an individual's appraisal of the degree to which situations in her/his life are overwhelming. Stress can lead to non-adherence to dietary recommendations and thus indirectly affect the patient's blood sugar. Due to stress, a person with diabetes may forget to take care of their food intake or take their medication, which will affect their blood sugar level. In general, stress is one of the factors increasing blood sugar level, leading to elevated mortality and prenatal complications in pregnancy. Pregnant women with gestational diabetes experience more stress than nond2iabetic pregnant women. 4 One of the most important components of gestational diabetes treatment is awareness, training, and recognition of patients' mental health problems. Counseling and education are an effective way to reduce the adverse effects of stress on the behavior of women with diabetes. Therefore, many psychological interventions are used simultaneously with medical interventions to manage the disease and its related complications. Mindfulness is one of the key components of the third wave of psychological treatment models. Mindfulness-based therapies are considered the third wave of cognitive therapy. Mindfulness meditation activates an area of the brain that creates positive emotions and beneficial effects on the body's immune function. As a result, events are perceived less distressing than they are at the moment, new thoughts are formed, and unpleasant emotions are reduced. 5 Previous studies reported the efficacy of mindfulness in reducing blood sugar level, stress, and depression and in increasing self-confidence in different groups of patients. 4 6 7 However, no study has yet investigated the effect of this intervention on the management of blood sugar in pregnant women. Women with gestational diabetes face many obstacles and challenges, including psychological stress, lack of information about the disease and its management, and fear of the disease. On the other hand, stress plays an important role in the onset, course, and prognosis of gestational diabetes and has adverse effects on blood sugar level in women with gestational diabetes. Therefore, the present study was performed to determine the effect of mindfulness-based stress reduction (MBSR) therapy on blood sugar level and stress in pregnant women with gestational diabetes who were under dietary treatment of diabetes and referred to urban health centers.

## Methods

The present educational intervention study was conducted to investigate the effect of MBSR counseling on the blood sugar and perceived stress of the patients. In this study, 80 pregnant women who were in the first half of pregnancy (< 20 weeks) and under treatment with gestational diabetes diet were included. These patients were selected among those who referred to health centers of Sirjan, Kerman province, Iran, provided that they met the inclusion criteria. Patients were excluded from the study due to pregnancy-related complications and none of the patients had to start pharmacological treatment. But 2 patients in the intervention group did not want to continue the counselling session so, finally, the study was performed on 78 patients.

### Inclusion Criteria

- Age between 18 and 45 years old;
- Being in the first half of pregnancy (given that the counseling procedure lasted for a month and there was a possibility of prescribing a medication or insulin by an endocrinologist and also to prevent serious complications induced by diabetes in the second half of pregnancy, we recruited patients who were in the first half of their pregnancy);
- Willingness to participate in the research;
- Singleton pregnancy;
- Being literate (at least elementary education);
- No history of gestational diabetes in previous pregnancies;
- No history of medical diseases (that is, cardiovascular and respiratory diseases) and pregnancy complications in previous pregnancies (such as preeclampsia, polyhydramnios, abnormal bleeding, placenta previa, recurrent miscarriage, giving birth to a high birthweight baby > 4 kg or fetal macrosomia, and stillbirth);
- No history of hospitalization in psychiatric department, use of psychotropic medications, and known personality and mental disorders;
- Suffering from gestational diabetes in the first half of pregnancy (fasting blood sugar [FBS] > 93 mg/dL or 2-hour PPBS > 120 mg/dL (75 g OGTT) according to national guidelines of the Ministry of Health).

### Discontinuation Criteria

- More than two absences in training sessions;
- Occurrence of diseases and complications related to pregnancy (such as cardiovascular, lung, and renal diseases, abortion, and preeclampsia) during the study;
- Starting drug treatment or insulin therapy in pregnancy;
- Occurrence of major stressful events during the research (new illness in spouse or children, death of a loved one, accident, migration, or bankruptcy);

Sample size was estimated to be 40 patients in each group according to a previous study, using sample size formula for two independent samples, taking into account the error of 5% and a power of 80%, and anticipating a 10% dropout rate.

### Measurements

To collect data, a demographic questionnaire and perceived stress scale developed by Cohen et al., were used. In addition, glucose kit (Pars Azmon product, made in Iran) was used to measure the blood sugar level of the patients.

### Demographic Questionnaire

The demographic questionnaire used in the present study extracted data on patients' age, body mass index (BMI), number of pregnancies, gestational age, birth-to-conception interval, education level, occupation, and the spouse's education level and occupation.

### Perceived Stress Scale Developed by Cohen

In the present study, the perceived stress scale developed by Cohen et al., was used to assess patients' perceived stress. This questionnaire has 14 questions, which are used to measure the general stress perceived in the past month. This questionnaire also assesses someone's thoughts and feelings about stressful events as well as their ability to control, overcome, and cope with such experienced stress. This questionnaire is suitable for determining the extent to which people recognize their stress in the face of unpredictable and uncontrollable life events. The questionnaire is scored based on a 5-point Likert scale (0  =  never, 1  =  almost never, 2  =  sometimes, 3  =  fairly often, 4  =  very often). Questions 4, 5, 6, 7, 9, 10, and 13 are scored in reverse (never = 4 to very often = 0). The minimum score is zero and the maximum score is 56. A higher score indicates greater perceived stress. 4 6

### Blood Glucose Measurement Kit and Test Basis

Pars Azmon kits were used to measure FBS and two-hour PPBS in the central laboratory of Sirjan. The glucose kit measures blood sugar using the enzymatic and colorimetric method (GOD-PAP) and does single point measurement using the photometric method.

### Procedure

**Fig. 1:**
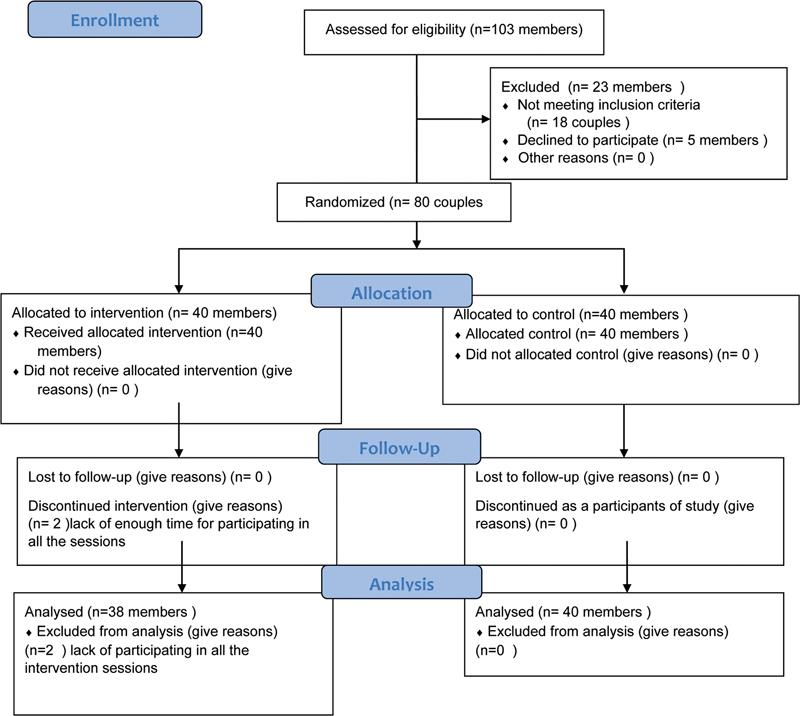
CONSORT Flow Diagram of participants.

After receiving ethics code (Kmu.ac.ir.1398.126), cluster sampling was done. For this purpose, out of 19 health centers, 6 centers were randomly selected as clusters by drawing lots. Then, from 6 centers, we randomly selected (by drawing lots) 3 centers as intervention group and 3 centers as control group. In the next step, among pregnant women with gestational diabetes who met the inclusion criteria, 40 were randomly assigned to each group. Gestational diabetes diagnosing was based on FBS and two-hour PPBS tests in pregnancy according to the national guidelines of the ministry of health of Iran. If the pregnant woman's FBS was > 93 mg / dL or blood sugar 2 hours after receiving 75 mg of glucose (two-hour PPBS) was > 120 mg / dL, she was diagnosed with gestational diabetes. For FBS and 2-hour PPBS tests (75g OGTT), two cc of blood was taken from pregnant women by the staff of Sirjan Central Laboratory. The FBS test was taken in the early morning and before eating breakfast. Two-hour PPBS test was done 2 hours after taking 75 g glucose. Then, FBS and two-hour PPBS were measured by laboratory kits using the ELISA method (Pars Azmon kits, Tehran, Iran). After receiving informed consent from each patient and assuring them about the confidentiality of their information, the study was initiated. Before intervention, both groups filled out the questionnaires. The MBSR sessions were held by one of the researchers, who had an MSc in counseling in midwifery and received mindfulness training exclusively. The intervention group underwent eight MBSR sessions in groups (each group consisted of 10 to 12 patients). The sessions were held twice a week and each session lasted for 90 minutes. During this period, the control group received only routine pregnancy care. The content of the training sessions is provided in chart 1 . One week after the intervention, the perceived stress scale (post-test) was completed by both groups. The patients' FBS and 2-hour PPBS were measured after the intervention. The study lasted 9 months from May, 2019 until January, 2021 ( Fig. 1 ).

**Chart 1:** Summary of sessions of counseling based on MBSR approach for reduction of Blood Sugar and Perceived-Stress in women with Gestational Diabetes Treated with diet

session	Content
1	Greeting and declaration of counseling rules, definition of the concepts of mindfulness and the main variables, description of the internal and external flow of the mind, eating raisins, homework.
2	Reviewing previous homework, mindful thinking, mindful examination of body and sitting meditation, homework.
3	Reviewing previous homework, focus on being present, practice seeing and hearing consciously in 3 minutes, focus on 5 senses in 5 minutes, homework.
4	Reviewing previous homework, stress and the body's reaction, practicing thoughts-emotions-body senses-behavior relationships, 3 minutes of concentration on an unpleasant event, mindful walking, homework.
5	Reviewing previous homework, effective responses to stress, 3-minute Breathing Space (3MBS), meditation in daily life, homework.
6	Reviewing previous homework, conscious mind interactions, take care of yourself, practicing speaking and listening consciously, practicing consecutive thoughts in an hour, getting feedback from participants from practicing, presenting homemade homework .
7	Reviewing previous homework, being more careful, mindful Yoga, making the unpleasant event enjoyable, providing homework
8	Reviewing previous homework, mountain meditation, summarization of all sessions, homework.

### Statistical Analysis

IBM SPSS Statistics for Windows, version 20 (IBM Corp., Armonk, NY, USA) was used for data analysis. Descriptive statistic such as frequency, mean, and percentage were used. Inferential statistics (independent samples *t* -test, paired *t* -test, chi-squared test, and the analysis of covariance) were also used. The statistically significant level was ≤ 0.05.

The present manuscript was derived from a master counselling in midwifery thesis (project code No. 97000964) and was approved by the Ethics Committee of Kerman University of Medical Sciences, Iran (code of ethics No. Kmu.ac.ir.1398.126). Written informed consent was obtained to enter the study and it was easy for participants to withdraw from the study whenever they were willing. At the request of the ethics committee, the study was conducted in accordance with the Declaration of Helsinki and Ethics Publication on Committee (COPE). Special codes were used for each of the participants to ensure the confidentiality of information.

## Results

The results showed that the mean age of the patients was 28.84 ± 6.20 in the intervention group and 29.03 ± 5.42 in the control group. In addition, the mean BMI of the patients in the intervention and control groups was 26.52 ± 5.38 and 26.12 ± 5.38, respectively. Two groups were homogeneous in terms of number of pregnancies, interpregnancy interval, own and spouse education level, and own and spouse occupation ( Table 1 ). According to Table 2 , there was a statistically significant difference between the two groups regarding the percentage of relative changes in stress, in a way that the intervention group experienced less perceived stress than the control group after the intervention ( *p* < 0.001).

**Table 1:** Demographic characteristics of participants

Group Variables		Intervention Mean ± SD	Control Mean ± SD	*p* - *value* *
Age		20.6 ± 84.28	29.03 ± 5.42	0.89
BMI		4.91 ± 52.6 2	26.12 ± 5.38	0.738
Variable		*n* (%)	*n* (%)	*p* - *value* **
Parity	First	13 (34.21)	9 (22.5)	0.317
	Second or more	25 (65.78)	31 (77.5)	
Distance from last pregnancy	0	13 (34.2)	10 (25)	0.428
	≤ 3 years	4 (10.52)	8 (20)	
	3 years≤	21 (55.26)	22 (55)	
Woman education	Diploma and less	22 (57.9)	22 (55)	0.756
	Associate Degree	3 (7.9)	2 (5)	
	Bachelor	12 (31.6)	13 (32.5)	
	Master's degree and higher	1 (2.6)	3 (7.5)	
Woman Job	Housekeeper	34 (89.5)	35 (87.5)	0.894
	Employed	4 (10.5)	5 (12.5)	
Spouse education	Diploma and less	21 (55.3)	22 (55)	0.98
	Associate Degree	3 (7.9)	4 (10)	
	Bachelor	12 (31.6)	12 (30)	
	Master's degree and higher	2 (5.3)	2 (5)	
Spouse Job	Self-employment	15 (39.5)	25 (62.5)	0.069
	Employee	23 (60.5)	15 (375)	

Abbreviations: BMI, body mass index; SD, standard deviation.

* T test.

** Chi-squared test.

**Table 2:** Comparison of relative changes in perceived stress after intervention in two groups

Group	Mean ± SD	Mann-Whitney Statistic	*p* - *value* *
Intervention	27.55 ± −49.19	- 7.23	< 0.001
Control	120.79 ± 57.35		

Abbreviation: SD, standard deviation.

* Mann-Whitney U test.

This means that after MBSR sessions, the amount of perceived stress was reduced in the intervention group. The results of the independent *t* -test for parametric data showed that the percentage of relative changes in FBS in the intervention and control groups was significantly different, and the intervention group had a higher decrease in the mean relative changes of FBS ( *p* = 0.02). In other words, after MBSR sessions, FBS decreased in the intervention group ( Table 3 ). Moreover, Mann-Whitney test results revealed that there was a significant difference between two groups with respect to the percentage of relative changes in FBS, and the mean FBS was lower in the intervention group than in the control group after the intervention. After the intervention, the mean 2-hour PPBS increased in the control group (20.85 ± 12.35) but decreased in the intervention group (27.35 ± - 3.5) ( *p* < 0.001). This means that 2-hour PPBS decreased in the intervention group after undergoing MBSR sessions ( Table 4 ).

**Table 3:** Comparison of relative changes in FBS after intervention in two groups

Group	Mean ± SD	Mann-Whitney Statistic	*p* - *value* *
Intervention	9.89 ± −11.46	- 2.34	0.02
Control	10.65 ± −6.01		

Abbreviation: SD, standard deviation

*Independent samples T Test.

**Table 4:** Comparison of relative changes BS after intervention in two groups

Group	Mean ± SD	Mann-Whitney Statistic	*p* - *value* *
Intervention	27.35 ± −3.5	- 3.759	< 0.001
Control	20.85 ± 12.35		

Abbreviation: SD, standard deviation

* Mann-Whitney U test.

## Discussion

According to the findings, MBSR was able to reduce stress in the intervention group. In this regard, Woolhouse et al. also showed that mindfulness-based intervention significantly reduced perceived stress in pregnancy. 8 In line with the present study, Guth et al. and Kiselev et al., showed that mindfulness-based training during pregnancy could effectively reduce pregnancy-related stress and anxiety. 9 10 Similarly, Beattie et al. reported that mindfulness-based training could help manage stressors, fear, and anxiety and regulate attention to be more present. 11 Mindfulness teaches us how to be aware of our thoughts and manage them. Mindfulness is not the cessation of thoughts since thoughts always arise in the mind. We become aware of own thoughts and their consequences through mindfulness, so we can manage them. To deal with physiological effects of stress, methods and programs called stress management have been proposed, including breathing exercises, relaxation techniques, meditation, and guided imagery. In the mindfulness technique, one turns one's mind from the past and the future to the present. When a person focuses on the present time, they see reality with all its internal and external aspects and realizes that the mind is constantly chewing on thoughts, ruminating on ideas, and talking internally because of the judgments and interpretations it makes. Practicing mindfulness gives a person the ability to realize that “thoughts are just thoughts” and when they realize that their thoughts may not be true, they can more easily let go of them, and consequently the perceived stress is reduced. Other findings of the study showed that MBSR could reduce FBS and 2-hour PPBS. Vieten et al. studied the effect of mindfulness-based training on stress and overeating during pregnancy. They found that mindfulness-based group training could help increase the skill in better management of stress and overeating during pregnancy. 12 Epel et al. also examined the effect of mindfulness-based intervention on anxiety, weight gain, and glycemic control in low-income pregnant women. They found that mindfulness-based intervention led to reduction of perceived stress and improvement of glucose tolerance. 13 The results of the present study also showed that MBSR counseling could be effective in reducing 2-hour PPBS in pregnant women with gestational diabetes through reducing stress and training techniques such as mindful eating, mindful seeing, and body scan. Various studies reported the efficacy of mindfulness-based counseling in controlling blood sugar and stress in diabetic patients, which confirms the findings of the present study. 4 14 15 Ni et al. conducted a systematic review and meta-analysis to investigate the effect of mindfulness-based intervention on blood glucose control and psychological outcomes in people with diabetes. In line with the findings of the present study, they concluded that mindfulness-based intervention had a significant effect on the reduction of HbA1c level and, as a result, control of blood glucose level in diabetic patients. 16 Although genetic factors have an important role in the etiology of diabetes, increasing prevalence of diabetes in recent decades is attributed to internal factors such as stress and external factors such as poor diet and low physical activity. In recent years, psychological aspects of diabetes are attended by the researchers. One of the methods for treating psychological problems in diabetic patients is mindfulness counselling. Research results in this field indicated the efficacy of this treatment in reducing stress as well as blood glucose level in people with diabetes. The extent of individual and family characteristics and individual motivations, which may affect the study, were beyond the control of the researcher. Due to holding counseling sessions twice a week, a feeling of tiredness and unwillingness to continue cooperation was observed in some cases. In this regard, a briefing session on the need to continue these sessions with the husbands of these pregnant women was conducted by phone.

## Conclusion

According to the results of the present study, stress and blood sugar level can be reduced by early intervention and provision of mindfulness counseling in women under treatment with gestational diabetes diet, especially in the first half of pregnancy. Since pregnant women are a vulnerable group in society and the rate of gestational diabetes is increasing, mental health and increasing the level of awareness on proper diet can be of particular importance in the health system.
